# Evaluating migraine tracking methods: a study of headache diary use in Lithuania

**DOI:** 10.3389/fneur.2026.1904398

**Published:** 2026-09-11

**Authors:** David Petrosian, Mantas Jokubaitis, Kristina Ryliškienė

**Affiliations:** 1 Faculty of Medicine, Vilnius University, Institute of Clinical Medicine, Clinic of Neurology and Neurosurgery, Vilnius, Lithuania; 2 Department of Nervous Diseases, Vilnius City Clinical Hospital, Vilnius, Lithuania; 3 Faculty of Medicine, Vilnius University, Vilnius, Lithuania

**Keywords:** diaries, headache, migraine, telehealth, telemedicine

## Abstract

**Introduction:**

Migraine management is essential for improving patient wellbeing. Headache diaries support active self-management and provide valuable clinical data to healthcare professionals. This study evaluated diary use, recording methods, and utilization patterns in Lithuania.

**Methods:**

In 2022–2023 an anonymous online survey was distributed nationwide through migraine support groups in social networks, online migraine self-help platforms, and the Lithuanian Association of Migraine Patients' website. Our questionnaire included sociodemographic, migraine-related, headache tracking methods, and previous experience with telemedicine.

**Results:**

A total of 847 patients were included in the study, with a median age of 35 years (IQR: 30–43); 823 (97.2%) participants were female. 345 (40.7%) patients reported using a headache diary, with 203 (58.8%) using paper-based, 167 (48.4%) electronic diaries, and 27 (7.8%) using both. Adherence to headache diaries was higher among chronic migraine patients compared to episodic migraine (76.3% vs. 38.1%, *P* < 0.001), participants receiving neurology telemedicine consultations compared to those without such experience (61.8% vs. 36.4%, *P* < 0.001), and among CGRP monoclonal antibody users relative to non-users (32.5% vs. 5.4%, *P* < 0.001). Use of migraine-tracking software declined with age (adjusted OR = 0.956, 95% CI 0.93–0.98, *P* < 0.001). Patients with chronic migraine were less likely to document attack-related information on electronic devices (adjusted OR = 0.531, 95% CI 0.28–0.98, *P* = 0.048). An inverse association was observed between migraine pain intensity during ineffective medication use and electronic tracking (adjusted OR = 0.877, 95% CI 0.76–0.99, *P* = 0.043). Physicians never reviewed headache diaries according to 12.8% of patients, while 13.6% reported that physicians were unaware of their diary use.

**Conclusions:**

This study provides insights into the preferences of Lithuanian migraine patients for tracking attack-related information. Younger patients and those with episodic migraine were more likely to use digital tools, while prior telemedicine experience was associated with headache diary use. These findings highlight how digital tools could be useful in managing migraines, while also showing that traditional paper-based approaches still have a place.

## 1 Introduction

Migraine is considered one of the most prevalent neurological disorders, significantly affecting and reducing the quality of life (1). Migraine management plays a crucial role in improving the wellbeing of patients. A headache diary allows patients to take a more active role in managing their condition, collecting valuable clinical data, and providing it to healthcare specialists (2, 3). Many patients use headache diaries not only to record attacks, but also to identify potential triggers, track patterns over time, and communicate their experiences more effectively with healthcare providers (3, 4). While the main function of migraine diaries is the same, they may vary in format and features (5). Although paper diaries remain a reliable option, electronic diaries are becoming more common and can offer valuable options, such as automated reminders, data visualization, and integration with medical records (6–8).

Despite the well-documented advantages of electronic diaries, the factors influencing their use for tracking migraine remain insufficiently understood, and little is known about the barriers to their widespread adoption. Traditional paper diaries are still widely used, although they have obvious disadvantages: dependence on manual entry and, above all, complex data analysis (9). Therefore, this study aimed to evaluate headache diary use, identify the methods employed to record migraine characteristics, and offer valuable insights into their utilization in Lithuania. We hypothesized that patients suffering from more severe forms of migraine and receiving more intensive and modern treatment are more likely to fill out a migraine diary.

## 2 Materials and methods

### 2.1 Survey structure and data collection

In this study, we conducted an anonymous online survey from 2022 to 2023. The questionnaire was developed by the study investigators based on the study objectives and relevant literature. No formal validation of the survey instrument was performed. All questionnaire items were mandatory. Submitted questionnaires contained no missing data. Responses were recorded only after survey submission, and participants who exited the survey before submitting their responses were not captured by the survey platform. Only completed questionnaires were included in the analysis. The questionnaire included: sociodemographic information (patients' age, gender, education, place of residence, employment status and type of work, self-reported digital literacy and presence of comorbidities), migraine-related data (the age of onset, type of migraine, pain intensity, number of monthly headache days (MHD), acute and preventive medications used, and monthly days with acute medications), headache diary features (the regularity of headache diary use, the methods utilized), and previous telemedicine experience, defined as having had a remote consultation with a physician before completing the survey. Medication overuse was not specifically assessed. Migraine diagnosis was based on participants' reported physician-confirmed diagnosis status. Migraine subtype was recorded according to participants' reported classification and was not independently verified using ICHD-3 criteria.

### 2.2 Study design

The designed survey was distributed nationwide through migraine support groups in social networks, online migraine self-help platforms, and the Lithuanian Association of Migraine Patients' website. Eligible participants were individuals with a self-reported physician-confirmed diagnosis of migraine who agreed to participate in the survey. Since the anonymous nature of data collection prevented the identification of individuals, ethical approval was not required according to the Vilnius Regional Committee on Biomedical Research Ethics, which adhered to Principle 26 of the General Data Protection Regulation.

### 2.3 Statistical analysis

Statistical analysis was conducted using R 4.3.2 software with statistical significance set at *P* < 0.05. For normally distributed data, means and standard deviations were reported, while non-normally distributed data were summarized using medians and interquartile ranges (IQR). When the variables were not normally distributed, we used the Mann–Whitney *U* test, otherwise, the *t*-test was employed. Chi-square and Fisher‘s exact were used to test for associations between categorical variables. Predictor variables included in the logistic regression models were selected based on their clinical relevance and potential association with headache diary use. Univariate and multivariate logistic regression analyses were performed to identify factors associated with the target variables.

## 3 Results

### 3.1 Population characteristics

A total of 1,046 survey responses were received. After excluding one respondent who refused participation and 198 without a diagnosed migraine, 847 respondents with migraine were included in the study. The median age of participants was 35 years (IQR 30–43), and 823 (97.2%) were female. The majority of migraineurs had higher education (*n* = 715, 84.4%) and lived in the capital or district center (*n* = 759, 89.6%). 345 patients (40.7%) reported headache diary use. Headache diary use was more common among patients with migraine without aura (*n* = 175, 50.7%) and chronic migraine (*n* = 45, 13.0%). Diary users reported more monthly headache days (median 6 days/month, IQR 4–10) and more days with acute medication use (median 6 days/month, IQR 4–10). They more frequently used triptans during attacks (*n* = 279, 80.9%) and were more likely to receive preventive treatment, including calcitonin gene-related peptide (CGRP) monoclonal antibodies (*n* = 112, 32.5%), antidepressants (*n* = 46, 13.3%), and beta blockers (*n* = 44, 12.8%, Table 1).

**Table 1:** Sociodemographic factors, migraine characteristics, and comorbidities of the study population.

Factors	Using a diary (*N* = 345)	Not using a diary (*N* = 502)	*P* value
Age, median (IQR)	35 (30–43)	35 (31–42)	0.409
Sex (female), *n* (%)	341 (98.8%)	482 (96.0%)	0.015
Living with a partner, *n* (%)	278 (80.6%)	432 (86.1%)	0.033
Having a child, *n* (%)	167 (48.4%)	305 (60.8%)	< 0.001
Acquiring private insurance, *n* (%)	134 (38.8%)	198 (39.4%)	0.860
Living in a capital/district center, *n* (%)	319 (92.4%)	440 (87.6%)	0.024
Self-reported digital literacy, median (IQR)	9 (8–10)	9 (8–10)	0.020^*^
Education			
Higher education, *n* (%)	294 (85.2%)	421 (83.9%)	0.594
Secondary education, *n* (%)	48 (13.9%)	78 (15.5%)	0.514
Employment			
White-collar work, *n* (%)	283 (82.0%)	419 (83.5%)	0.585
Blue-collar work, *n* (%)	81 (23.4%)	108 (21.5%)	0.500
Student, *n* (%)	32 (9.3%)	32 (6.4%)	0.117
Retired, *n* (%)	2 (0.6%)	6 (1.2%)	0.363
Unemployed, *n* (%)	19 (5.5%)	22 (4.4%)	0.454
Remote work, *n* (%)	90 (26.1%)	115 (22.9%)	0.289
In-person work, *n* (%)	255 (73.9%)	387 (77.1%)	0.289
Age of migraine onset, median (IQR)	18 (14–25)	18 (14–25)	0.933
Migraine type and characteristics			
Migraine with aura, *n* (%)	110 (31.9%)	188 (37.5%)	0.096
Migraine without aura, *n* (%)	175 (50.7%)	135 (26.9%)	< 0.001
Chronic, *n* (%)	45 (13.0%)	14 (2.8%)	< 0.001
Vestibular, *n* (%)	9 (2.6%)	10 (2.0%)	0.552
Menstrual, *n* (%)	41 (11.9%)	50 (10.0%)	0.374
Average migraine pain when medications are ineffective, median (IQR)	8 (7–9)	8 (7–9)	0.182
Monthly headache days, median (IQR)	6 (4–10)	5 (3–8)	< 0.001
Missed workdays due to migraine per month, median (IQR)	0 (0–1)	0 (0–1)	0.149
Frequency of acute medication use (days/month), median (IQR)	6 (4–10)	5 (3–8)	< 0.001
Medication usage during migraine attacks, *n* (%)	338 (98.0%)	478 (95.2%)	0.036
Combined analgesics with codeine and/or caffeine, *n* (%)	104 (30.1%)	176 (35.1%)	0.135
NSAIDs, *n* (%)	270 (78.3%)	400 (79.7%)	0.617
Triptans, *n* (%)	279 (80.9%)	276 (55.0%)	< 0.001
Preventive migraine treatment, *n* (%)	163 (47.2%)	58 (11.6%)	< 0.001
Antidepressants, *n* (%)	46 (13.3%)	15 (3.0%)	< 0.001
Beta blockers, *n* (%)	44 (12.8%)	37 (7.4%)	0.009
CGRP monoclonal antibodies, *n* (%)	112 (32.5%)	27 (5.4%)	< 0.001
OnabotulinumtoxinA, *n* (%)	6 (1.7%)	4 (0.8%)	0.592
Antiepileptic medications, *n* (%)	10 (2.9%)	4 (0.8%)	0.018
Usage of medications for comorbidities, *n* (%)	129 (37.4%)	160 (31.9%)	
Cardiovascular diseases, *n* (%)	52 (15.1%)	74 (14.7%)	0.894
Endocrine diseases, *n* (%)	21 (6.1%)	32 (6.4%)	0.865
Gastrointestinal diseases, *n* (%)	37 (10.7%)	83 (16.5%)	0.017
Other neurologic or psychiatric diseases, *n* (%)	58 (16.8%)	52 (10.4%)	0.006

* Despite identical medians with interquartile ranges, the mean values differed significantly (*p* < 0.05).

IQR, interquartile range; NSAIDs, nonsteroidal anti-inflammatory drugs; CGRP, calcitonin gene-related peptide.

Among diary users, 203 (58.8%) preferred paper-based diaries, 167 (48.4%) preferred electronic diaries, and 27 (7.8%) used both formats. The most common option for attack recording in the electronic sample was using the Migraine Compass app (*n* = 70, 41.9%). In contrast, most of those who used paper diaries followed a template provided by the National Migraine Society (*n* = 60, 29.6%). Survey participants reported that tracking migraine attacks was primarily recommended by neurologists (*n* = 256, 74.2%). Most migraineurs recorded headache data on the same day as the attack (*n* = 220, 63.8%). Physician review of this data was reported as occurring always (n = 169, 49.0%), usually (*n* = 85, 24.6%), or never (*n* = 44, 12.8%). Detailed results are presented in Table 2.

**Table 2:** Headache diary types, usage habits, and physician involvement.

Factors	Using a diary (*n* = 345)
**Diary type** ^*^	
Paper, *n* (%)	203 (58.8%)
Electronic, *n* (%)	167 (48.4%)
**Paper diary format**	
Paper-based calendar diary, *n* (%)	84 (41.4%)
Template from the National Migraine Society, *n* (%)	60 (29.6%)
Self-created notes for migraine tracking, *n* (%)	44 (21.7%)
Template given by the physician, *n* (%)	22 (10.8%)
Template from another specialized headache website, *n* (%)	6 (3.0%)
App usage	
Migraine compass, *n* (%)	70 (41.9%)
Migraine buddy, *n* (%)	46 (27.5%)
Default phone calendar, *n* (%)	14 (8.4%)
Notes, *n* (%)	9 (5.4%)
Flo, maya, woman log, *n* (%)	6 (3.6%)
Manage my pain, *n* (%)	3 (1.8%)
Headache log, *n* (%)	2 (1.2%)
Recommended filling out a diary	
Neurologist, *n* (%)	256 (74.2%)
Oneself, *n* (%)	67 (19.4%)
General practitioner, *n* (%)	11 (3.2%)
Others, *n* (%)	11 (3.2%)
Filling regularity	
During headache on the same day, *n* (%)	220 (63.8%)
By recall on pain-free days, *n* (%)	102 (29.6%)
Every day, *n* (%)	23 (6.7%)
Physician reviews headache diary during consultation	
Always, *n* (%)	169 (49.0%)
Usually, *n* (%)	85 (24.6%)
Physician unaware of diary being filled out, *n* (%)	47 (13.6%)
Never, *n* (%)	44 (12.8%)

^*^Participants could select more than one migraine-tracking method.

### 3.2 Determinants of headache diary and electronic diary use

Among the factors associated with increased likelihood of headache diary use were CGRP monoclonal antibody use [OR = 6.596, 95% CI (4.06–11.04), *P* < 0.001]. Having chronic migraine significantly increased the likelihood of headache diary use [OR = 2.962, 95% CI (1.40–6.63), *P* = 0.006]. Our results also suggest that prior experience with telemedicine was positively associated with diary use [OR = 1.687, 95% CI (1.21–2.36), *P* = 0.002]. Other factors associated with headache diary use are listed in Table 3.

**Table 3:** Logistic regression analysis of factors associated with headache diary use.

Factor	Univariate logistic regression			Multivariate logistic regression		
	OR	**95% CI**	***P*** **value**	**OR**	**95% CI**	***P*** **value**
Age	0.992	0.98–1.01	0.269	-	-	-
Sex (female)	3.541	1.32–12.24	0.022	8.385	2.45–35.75	< 0.002
Education (higher)	1.112	0.76–1.63	0.594	-	-	-
Living in a capital/district center	1.731	1.08–2.84	0.025	2.081	1.19–3.75	0.012
Having a child	0.613	0.46–0.80	< 0.001	0.631	0.45–0.87	0.006
Digital literacy	1.122	1.02–1.23	0.023	1.195	1.07–1.34	0.003
Experience in telemedicine	2.334	1.75–3.12	< 0.001	1.687	1.21–2.36	0.002
Chronic migraine	5.232	2.89–10.03	< 0.001	2.962	1.40–6.63	0.006
Monthly headache days	1.053	1.02–1.08	< 0.001	1.011	0.98–1.05	0.535
Migraine without aura	2.801	2.10–3.74	< 0.001	2.171	1.55–3.04	< 0.001
Migraine pain intensity when medications are ineffective	1.072	0.98–1.16	0.124	-	-	-
CGRP monoclonal antibodies	8.463	5.48–13.48	< 0.001	6.596	4.06–11.04	< 0.001
Antiepileptic medications	3.724	1.23–13.64	0.028	5.531	1.55–23.27	0.012
Triptans	3.462	2.52–4.80	< 0.001	1.966	1.37–2.84	< 0.001
Beta blockers	1.843	1.16–2.92	0.010	1.264	0.73–2.19	0.402

Younger patients were more likely to use software for migraine tracking, with lower use observed at higher ages (OR = 0.956, 95% CI 0.93–0.98, *P* < 0.001). Patients with chronic migraine were less likely to fill out the attack-related information on electronic devices [OR = 0.531, 95% CI (0.28–0.98), *P* = 0.048]. The inverse association was found with the migraine pain intensity when medications are ineffective [OR = 0.877, 95% CI (0.76–0.99), *P* = 0.043]. Other evaluated factors are presented in Table 4 and were not significantly associated with electronic headache diary use.

**Table 4:** Logistic regression analysis of factors associated with electronic headache diary use.

Factors	Univariate logistic regression			Multivariate logistic regression		
	**OR**	**95% CI**	***P*** **value**	**OR**	**95% CI**	***P*** **value**
Age	0.954	0.93–0.98	< 0.001	0.956	0.93–0.98	< 0.001
Sex (female)	0.420	0.21–3.32	0.454			
Education (higher)	0.755	0.40–1.42	0.378	-	-	-
Living in a capital/district center	0.706	0.31–1.61	0.405	-	-	-
Having a child	0.892	0.57–1.39	0.613	-	-	-
Digital literacy	1.130	0.96–1.33	0.136	-	-	-
Experience in telemedicine	1.170	0.75–1.83	0.490	-	-	-
Chronic migraine	0.482	0.23–0.96	0.045	0.531	0.28–0.98	0.048
Monthly headache days	0.984	0.94–1.03	0.433	-	-	-
Migraine without aura	0.918	0.59–1.43	0.704	-	-	-
Migraine pain intensity when medications are ineffective	0.861	0.75–0.99	0.035	0.877	0.76–0.99	0.043
CGRP monoclonal antibodies	0.756	0.47–1.21	0.250	-	-	-
Antiepileptic medications	1.716	0.37–8.83	0.485	-	-	-
Triptans	0.970	0.56–1.69	0.914	-	-	-
Beta blockers	0.809	0.40–1.60	0.548	-	-	-

### 3.3 Trends in diary usage among migraine groups and the presence of comorbidities

It was found that chronic migraine patients filled out a headache diary more frequently compared to the episodic group (76.3% vs. 38.1%, *P* < 0.001). In addition, chronic migraine patients preferred paper-based approaches significantly more (73.3% vs. 56.7%, *P* = 0.034). A comparative analysis between patients with comorbidities and those without showed that paper diaries were preferred more by the comorbid group (70.5% vs. 51.9%, *P* < 0.001), while electronic-based approaches were more frequently used by patients without concomitant conditions (38.0% vs. 54.6%, *P* = 0.003). Statistical analysis revealed no significant differences in the regularity of filling or the use of the apps between the above-mentioned samples.

### 3.4 Impact of telemedicine experience on diary use

The findings strongly indicate that telemedicine was associated with headache diary use (Table 5). Patients who received remote consultations from neurologists were more likely to record their attack-related data compared to those who did not engage in telemedicine (61.8% vs. 36.4%, *P* < 0.001). The odds ratio for completing a diary was 2.83 [95% CI (1.96–4.11), *P* < 0.001], highlighting a strong positive association with remote consultations. Additionally, the results revealed that prior telemedicine consultations with neurologists were associated with the choice of applications for documenting migraine.

**Table 5:** Association between teleconsultation and headache diary-filling patterns.

Variable	Was consulted by a neurologist remotely (*N* = 144)	Was not consulted by a neurologist remotely (*N* = 703)	Univariate logistic regression analysis		
			**OR**	**95% CI**	**P value**
Filling a diary	89 (61.8%)	256 (36.4%)	2.83	1.96–4.11	< 0.001
**Diary type**					
Paper, *n* (%)	55 (61.8%)	148 (57.8%)	1.18	0.72–1.95	0.511
Electronic, *n* (%)	47 (52.8%)	120 (46.9%)	1.27	0.78–2.06	0.336
App usage					
Migraine buddy	12 (25.5%)	34 (28.3%)	1.02	0.48–2.02	0.962
Migraine compass	28 (59.6%)	42 (35.0%)	2.34	1.33–4.07	0.003

## 4 Discussion

In our study, fewer than half of migraine patients reported using a headache diary, with paper diaries being used more often than electronic tools. Use of headache diaries was more frequent in patients with chronic migraine, with prior telemedicine experience, and among those receiving preventive treatment, particularly CGRP monoclonal antibodies. This suggests that diary recording was used not only to assess migraine symptoms but also to evaluate the effectiveness of preventive treatment, and it was linked to the legal framework governing the prescription of reimbursed medications (triptans and, in particular, CGRP monoclonal antibodies). This may explain our finding, that diary recording was most often initiated by neurologists, and the role of general practitioners was limited—only 3.2% of patients reported that their general practitioner encouraged them to start monitoring their headaches.

Given the rapid technological advancements across all aspects of the healthcare system, our findings indicate that patients are open to exploring innovative tools for migraine management (10). Our research showed that 48.4% of migraine patients completed an electronic diary, reflecting a lower degree of engagement than a study conducted in the United Kingdom by Jonker et al., with a larger rate of digital tracking tool acceptance (82%) (11). This tendency can be influenced by cultural attitudes toward digital health, individual preferences, and overall familiarity with digital devices. It is not surprising that electronic diaries are more commonly used among younger patients. Such association may be influenced by younger generations' ability to adapt to and cope with innovative technologies more quickly.

In contrast, older patients and those with chronic migraine, as it was found in our study, or comorbid conditions opt for a more conservative, paper-based approach. This preference may reflect the simplicity of paper diaries and the difficulties of using digital tools during severe headache episodes. Despite applications being designed to be user-friendly (12), factors such as persistent headache, cognitive and physical fatigue, and light sensitivity may make their use difficult. Additionally, prolonged screen exposure has been associated with eye strain and visual discomfort (13, 14), which may represent an additional consideration when evaluating the usability of digital tools in patients with migraine. These patients often prefer paper diaries because digital tools can feel complicated or uncomfortable to use. Therefore, paper diaries remain a more suitable option for documenting attack-related information in certain patient groups.

Previous studies have shown that migraineurs prefer diaries that are low-effort to use and easy to interpret. Trackers that require heterogeneous data can eventually produce sophisticated visual or textual outputs, but this may place a higher cognitive load on patients' self-care, and the results may remain unclear to them. For this reason, patients tend to show their statistics to physicians to gain deeper insight into the diary results. In addition, it is also important for patients to be able to add free-text entries to better understand context and triggers. In contrast, clinicians primarily need structured, reliable data to guide diagnosis and treatment. The development of user-friendly, easy-to-use trackers is important to encourage patient engagement with apps, reduce skipped entries, and support better clinical communication (15–17). Our findings suggest that physicians were unaware of headache diary use in 13.6% of cases, and 12.8% of patients reported that their diaries were never reviewed during consultations. This suggests that headache diaries are not consistently integrated into routine clinical practice, possibly because of limited consultation time or the absence of standardized workflows for reviewing patient-generated data. This may limit the clinical value of headache diaries. When it comes to electronic diaries, including them in medical records could make migraine care more effective by making patient information easier to access, but data privacy and security still need to be protected (18, 19). Future studies should investigate whether routine physician review of headache diaries improves treatment outcomes.

Our study shows that previous telemedicine consultations were associated with a higher likelihood of headache diary recording. Patients who underwent remote consultations with neurologists appeared more committed to tracking their migraine attacks. This suggests that telemedicine experience may be associated with greater engagement in self-management among patients with migraine. Alternatively, it is possible that patients who are more comfortable with digital technologies, such as mobile apps, are also more likely to prefer telemedicine services. We found that patients with prior telemedicine experience were significantly more likely to use the Migraine Compass app demonstrating a strong association between remote consultations and the use of the application. Compared with Migraine Buddy, Migraine Compass supports the official national language, which makes it easier for patients to use the app. This shows that language and culture matter when designing digital tools. Consequently, to ensure broader adoption, developers should proactively address language barriers (20).

A strength of this study is the large sample size, which was collected from respondents across the entire country. Also, our study highlights the role of sociodemographic factors and migraine-related characteristics in headache diary use.

A limitation of this study is the reliance on self-reported migraine diagnosis and subtype, which were not independently verified according to ICHD-3 criteria. In addition, recruitment through migraine support groups and patient organizations may have introduced selection bias by attracting patients who were more engaged in migraine self-management. Moreover, a significant part of our sample consisted of residents of capital cities or urban centers, which is likely related to a higher level of technological familiarity (21). These factors might limit how well the findings apply to the wider population. Future research should include a more diverse sample and consider socioeconomic variables to provide a deeper understanding of the barriers to digital diary use.

## 5 Conclusion

Our study provides valuable insights into migraineurs' preferences for recording attack-related information in Lithuania. Almost half of the patients in this study used electronic diaries, although paper-based methods remained the most common. Younger patients and those with episodic migraine were more likely to use digital tools, and previous experience with telemedicine was associated with headache diary use. These findings highlight the potential for expanding digital solutions in migraine management while also acknowledging the ongoing importance of traditional methods.

## Data Availability

The datasets presented in this article are not readily available because they contain anonymized survey data collected from human participants and are subject to ethical and data protection considerations. Requests to access the datasets should be directed to David Petrosian, david.petrosian@santa.lt.
