# Supplementary material for: Evaluating migraine tracking methods: a study of headache diary use in Lithuania
Source: Front Neurol. 2026 Sep 11;17:1904398. doi: 10.3389/fneur.2026.1904398 (PMC13612321; doi:10.3389/fneur.2026.1904398)
Supplement: Supplementary file 1 [file Data_Sheet_1.pdf]

1. **I confirm that I voluntarily consent to participate in this study and agree that my anonymous responses may be used for scientific research**

Yes

No

2. **Age (enter a number):**
3. **Sex:**

Female

Male

Other

4. **Education:**

Incomplete secondary education

Secondary / vocational education

Higher education

5. **Place of residence:**

Capital / district center

Other:

6. **In which county do you live?**

Vilnius

Kaunas

Klaipėda

Marijampolė

Panevėžys

Šiauliai

Tauragė

Telšiai

Utena

Alytus

7. **Work activity (indicate all applicable options):**

School student / University student

I do physical work

I do mental work

Unemployed

Retired person

8. **Work style:**

Most of the work is remote work

Most of the work is in-person work

9. **Marital status:**

I live alone

I live with a partner/spouse

10. **I have a preschool/school-age child/children:**

Yes

No

11. **Assess your digital literacy: (0 – I have no skills, 1–3 – basic knowledge, 4-7 - average knowledge, 8-9 - advanced knowledge, 10 - proficient user)**

0 1 2 3 4 5 6 7 8 9 10

12. **I have private health insurance:**

Yes

No

13. **I usually go to the healthcare facility:**

By car

Public transport

Taxi

Non-motorized transport (bicycle, scooter)

On foot

Other:

14. **What distance do you live from the healthcare center where a family doctor consultation is provided (enter the distance in kilometres)?**
15. **What distance do you live from the healthcare center where a neurologist consultation is provided (enter the distance in kilometres)?**

16. **Do you regularly take medication for other illnesses?**

Yes

No

17. **For what other illnesses are you taking medication (select all that apply)?**

Neurological/mental health disorders

Diseases of the cardiovascular system

Diseases of the digestive system

Respiratory system diseases

Urinary tract diseases

Gynecological diseases

Muscle/joint diseases

Skin diseases

Oncological diseases

I do not take medications for other illnesses

Other:

18. **Have you been diagnosed with migraine?**

Yes

No

19. **At what age did the migraine attacks begin (enter a number)?**
20. **What type of migraine have you been diagnosed with (Select all that apply)?**

Migraine with aura

Migraine without aura

Chronic

Vestibular migraine

Menstrual migraine

I have been diagnosed with migraine, but I do not know which type

I have not been diagnosed with migraine

21. **What is the average severity of your migraine when pain medications are ineffective? 0 – no headache; 4–5 – moderate pain; 10 – the worst imaginable pain**

0 1 2 3 4 5 6 7 8 9 10

22. **How many days per month do you have headache (enter a number)?**
23. **How many days were you absent from school/work during the past month because of migraine (enter a number)?**
24. **Do you take pain medication during a migraine attack?**

Yes
No

25. **How many days per month do you take headache medication (enter a number)?**
26. **What headache medications have you taken over the past year (select all that apply)?**

Aspirin

Paracetamol

Other nonsteroidal anti-inflammatory drugs (e.g., ibuprofen, diclofenac, etc.)

Combination analgesics with caffeine or codeine (e.g., Citramon, Saridon, Askofen, Solpadeine)

Triptans (e.g., sumatriptanum (Imigran, Cinie), frovatriptanum (Migard), rizatriptanum (Maxalt), zolmitriptanum (Zomig), naratriptanum (Naramig, Naratriptan), almotriptanum)

Intramuscular medications (e.g., diclofenacum (Naklofen), ketorolacum (Ketanov), dexketoprofen (Dolmen), metamizole (Analgin), metoclopramide (Cerucal), dexamethasonum)

Sedative medications (e.g., bromazepam (Lexotanil), alprazolam (Xanax), diazepam (Relanium), etc.)

Other:

27. **Are you currently using preventive medication for migraine, for example amitriptyline, propranolol, Aimovig, Betaloc, Ajovy, Botox, etc.?**

Yes

No

28. **What migraine preventive treatment are you using (select all that apply)?**

Beta blocker: metoprolol (Betaloc, Corvitol, Emzok) / propranolol

Antiepileptic medications: valproic acid (Depakine Chrono) / topiramate (Topamax)

Antidepressants: amitriptyline / venlafaxine (Alventa, Lanvexin, Efexor) / nortriptyline (Noritren)

ACE-inhibiting medications: candesartan (Candesartan, Canocombi, Canocord) / lisinopril (Dironorm, Diroton)

Botulinum toxin (Botox), injections were administered by a neurologist (31–39 injections during one procedure)

Botulinum toxin (Botox, Dysport), injections performed by a beauty specialist or a physician who is not a neurologist (several injections in the forehead area)

Erenumab (Aimovig), Fremanezumab (Ajovy), Galcanezumab (Emgality)

I do not use preventive treatment

29. Have you been filling in a headache diary or calendar during the past 1 year?

Yes Go to question 30

No Go to question 37

30. Do you regularly keep a headache diary?

I always fill it out daily

I am filling it out only during a headache on the same day

I am filling it out from memory on days when I do not have pain

31. Who recommended filling out the diary?

Neurologist

General practitioner

Other patients suffering from migraine

I decided myself

Other:

32. Which diary do you fill out (select all that apply)?

Paper form

Electronic

Other:

33. Which paper calendar do you fill out (select all that apply)

I print from sergumigrena.lt

I print it out from another specialized headache website

I draw it myself

I use a simple paper calendar

I do not fill it out

Other:

34. Which mobile application do you use (select all that apply)?

Migraine Buddy

Manage My Pain

Migraine Insight

Migraine Monitor

Migraine Compass

Headache Log

I do not use it because I did not know that such an option exists

I do not use it because a paper diary is more convenient for me

Other:

35. Is there a lack of a mobile app for monitoring migraine attacks with advice and recommendations in Lithuanian?

Yes

No

36. Does the physician review your headache diary during the consultation?

Always

Usually

Never

My physician does not know that I am filling out a headache diary

37. Have you ever had a remote consultation with a neurologist regarding headache?

Yes Go to question 38

No, because my treating neurologist does not offer remote consultations

Go to question 58

No, I did not inquire whether the neurologist consults remotely

Go to question 58

No, because I prefer not to have remote consultations

Go to question 58

38. **Was the remote consultation an initial consultation (i.e., the first time you contacted a neurologist regarding headache) or a follow-up consultation?**

Initial

Follow-up

39. **How many times have you had a remote consultation? (enter a number)**
40. **How did you register for the remote consultation (select all that apply)?**

Online through the information system

By phone

In person at the healthcare center

By email

Other:

41. **What inconveniences did the registration for the remote consultation cause (select all that apply)**

Difficulty getting through to the reception desk

Lack of available appointments

The registration system was not user-friendly

Frequent disruptions of the registration system

I did not notice any discomfort.

Other:

42. **Were you able to choose the consultation method (telephone consultation, video call)?**

Yes

No

43. **What type of consultation took place (select all that apply):**

By phone

Video call

Other:

44. **Would you like remote consultations to be conducted by video call more often?**

Yes

No

45. **Average waiting time between registration and the remote consultation date (enter the number of days):**
46. **Did the remote consultation take place at the scheduled time?**

Consultation took place on time

<5 min. late

5–10 min. late

10–30 min. late

>30 min. late

Earlier than scheduled

47. **Most common average duration of a remote consultation (enter the duration in minutes):**
48. **Was the remote consultation paid for?**

No

Yes, the price is the same as an in-person consultation

Yes, the price is lower than an in-person consultation

Yes, the price is higher than an in-person consultation

49. **Was a new headache diagnosis established during the remote consultation?**

Yes

No

50. Was a new treatment for headache attacks prescribed during the remote consultation?

Yes

No

51. Was the newly prescribed acute treatment effective?

Yes

No

No new treatment was prescribed

52. Was preventive migraine medication prescribed during the remote consultation?

Yes

No

53. Was the newly prescribed preventive treatment effective?

Yes

No

No new treatment was prescribed

54. Was a referral to the emergency department and urgent care given during the remote consultation?

Yes

No

55. Was the previously used treatment continued during the remote consultation?

Yes

No

56. During the remote consultation, was a sick leave certificate issued due to headache?

Yes

No

57. **Overall, how would you rate your satisfaction with the remote consultation provided by a neurologist regarding headache?**

0 1 2 3 4 5 6 7 8 9 10

58. **Have you ever had a remote consultation with a general practitioner due to headache?**

Yes Go to question 59

No

59. **Was the remote consultation initial (i.e., first time contacting the family doctor because of a headache) or follow-up?**

Initial

Follow-up

60. **How many times have you had a remote consultation? (enter a number)**
61. **How did you register for the remote consultation? (select all that apply)**

Online through the information system

By phone

In person at a healthcare center

By email

Other:

62. **What inconveniences did the registration for the remote consultation cause (select all that apply)**

Difficulty contacting the reception desk

Lack of available appointments

The registration system was not user-friendly

I did not notice any discomfort

Other:

63. **Were you able to choose the consultation method (telephone consultation, video call)?**

Yes

No

64. What type of consultation took place (select all that apply):

By phone

Video call

Other:

65. Would you like remote consultations to be conducted by video call more often?

Yes

No

66. Average waiting time from registration to the day of the remote consultation (number of days):

67. Did the remote consultation(s) take place at the scheduled time?

Consultation took place on time

<5 min. late

5–10 min late

10–30 min. late

>30 min. late

Earlier than scheduled

68. Most common average duration of a remote consultation (enter the duration in minutes):

69. Was the remote consultation paid for?

No

Yes, the price is the same as an in-person consultation

Yes, the price is lower than an in-person consultation

Yes, the price is higher than an in-person consultation

70. Was a new diagnosis established during the remote consultation?

Yes

No

71. **Was a new treatment prescribed during the remote consultation?**

Yes

No

72. **Was the treatment effective?**

Yes

No

New treatment was not prescribed

73. **Was a referral to the emergency department and urgent care given during the remote consultation?**

Yes

No

74. **Was previously used treatment continued during the remote consultation?**

Yes

No

75. **Was a sick leave certificate issued due to headache during the remote consultation?**

Yes

No

76. **Overall, how would you rate your satisfaction with the remote consultation provided by your general practitioner regarding headache?**

0 1 2 3 4 5 6 7 8 9 10
